# Ophthalmoscopic Course for Army Veterinarians

**Published:** 1898-07

**Authors:** 


OPHTHALMOSCOPIC COURSE FOR ARMY VETERINARIANS. According to the regulations of the Austrian Ministry of War, two ophthalmoscopic courses, one at the Imperial Military Veterinary Institute and the other at the Veterinary High School, are given this year for the benefit of army veterinarians. These courses last about three weeks and are given between the 1st and 21st of May and the 1st and 21st of June. The hearers were some twenty army veterinarians connected with the cavalry, and ten took part in each course. The War Ministry has provided the necessary instruments of investigation, consisting of an ophthalmoscope and a Priestley-Smith lamp for the veterinarian of each regiment. In order to provide sufficient material for practice the War Ministry has given these veterinarians access to the various clinics and allowed them to examine any number of the horses belonging to the Vienna cavalry and field artillery. These regulations have been gladly received in veterinary circles as a means of developing along special lines.—*Idem.*

